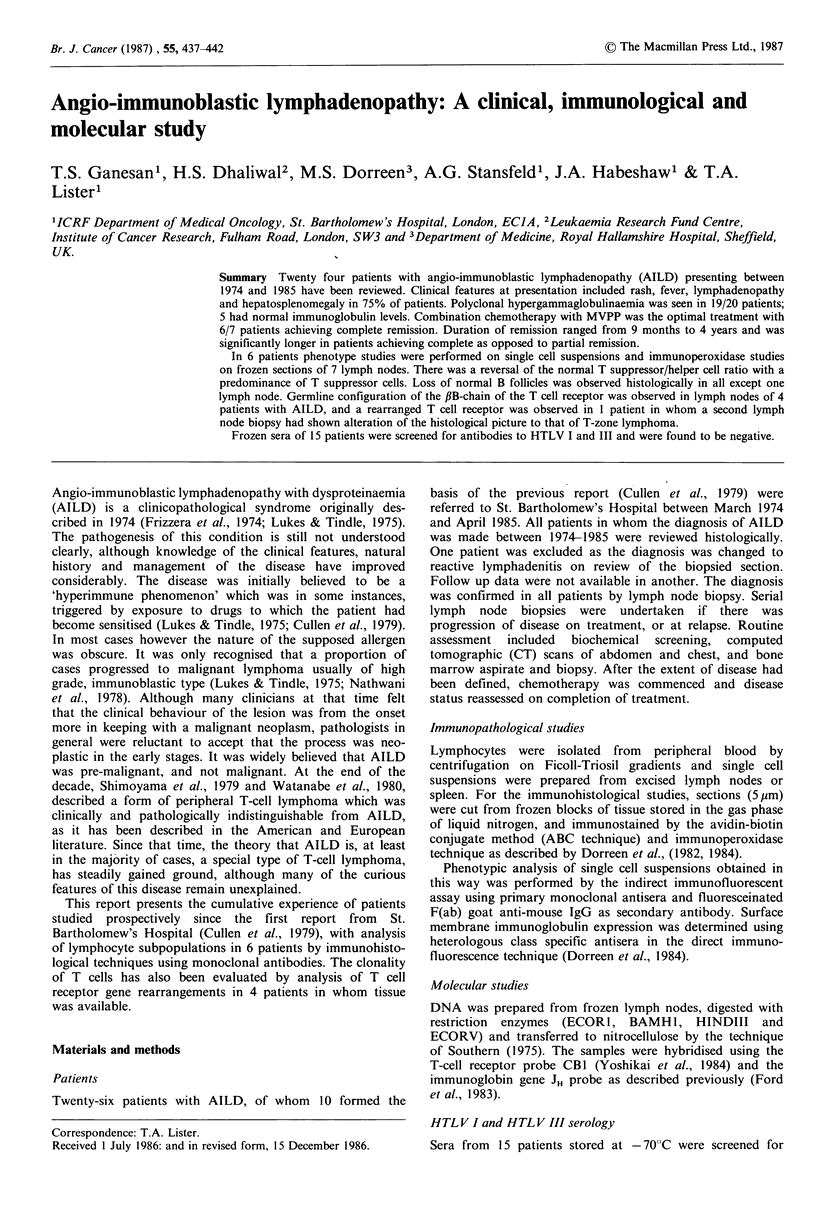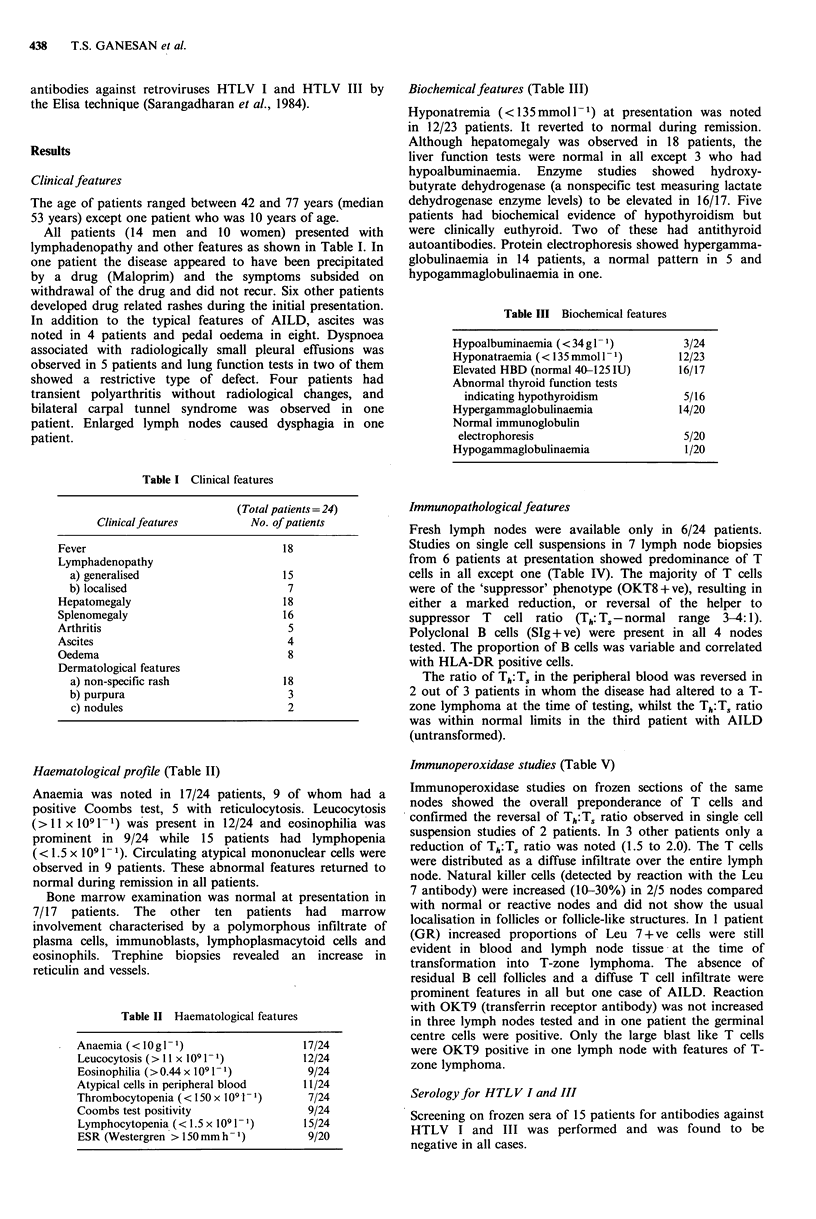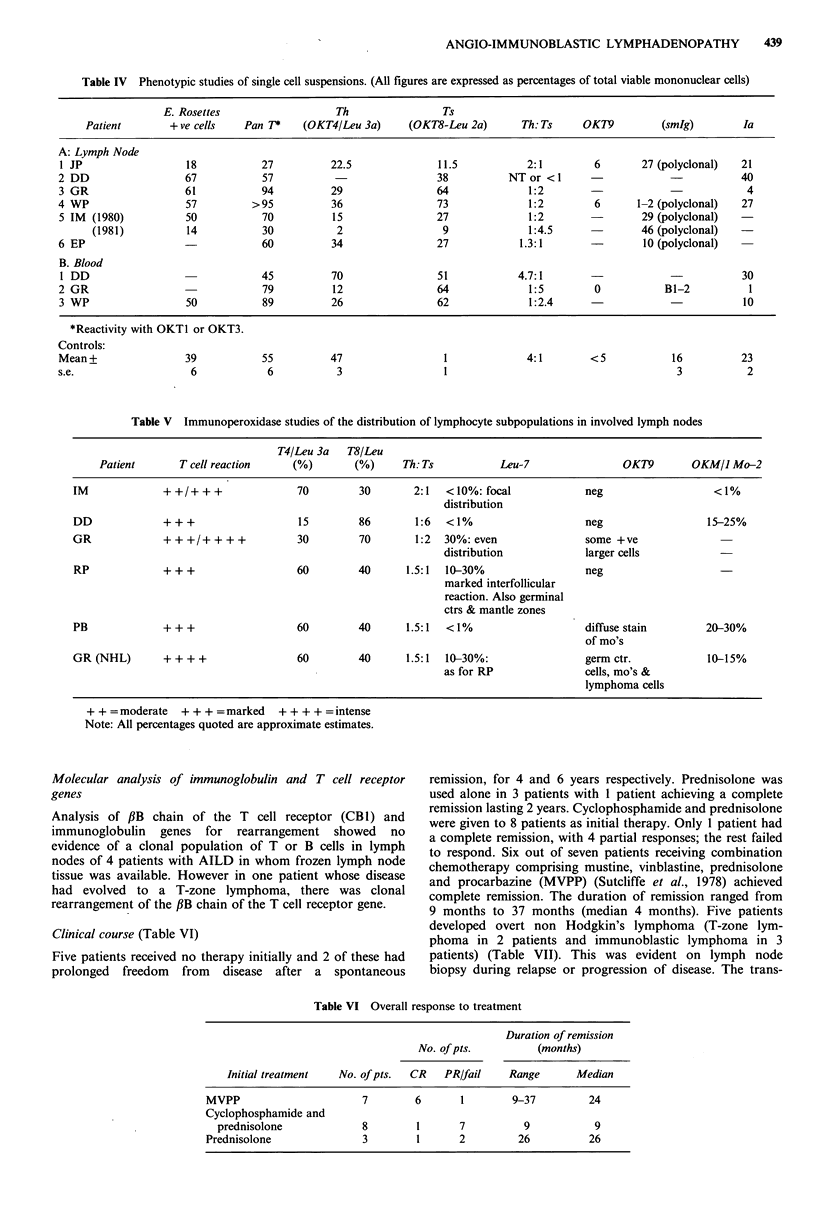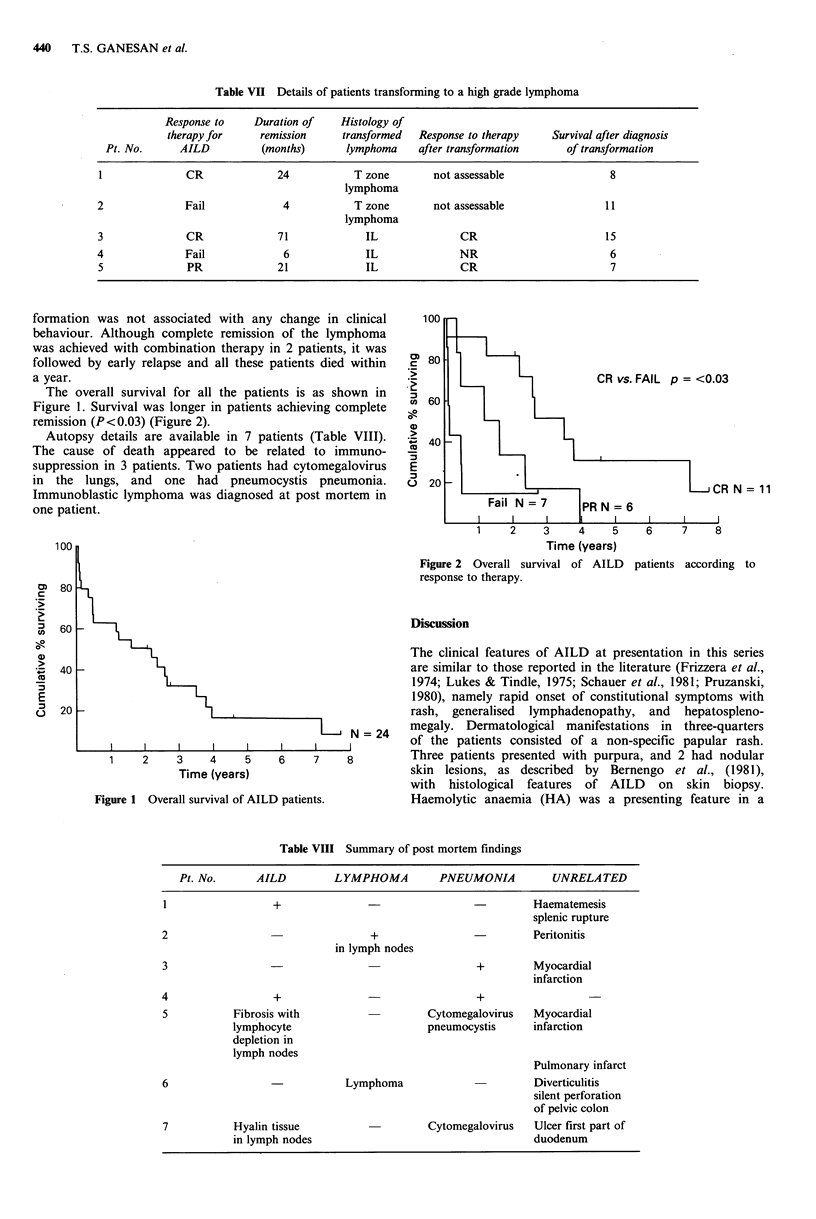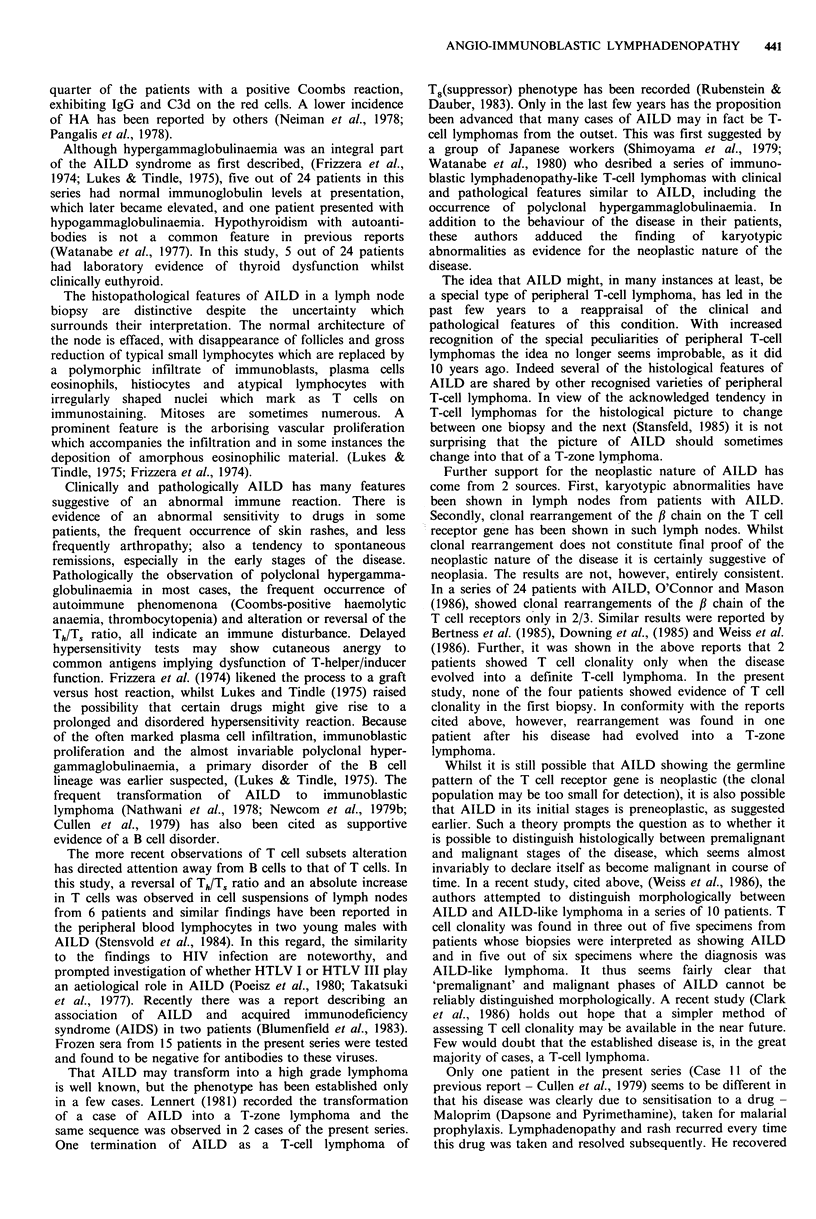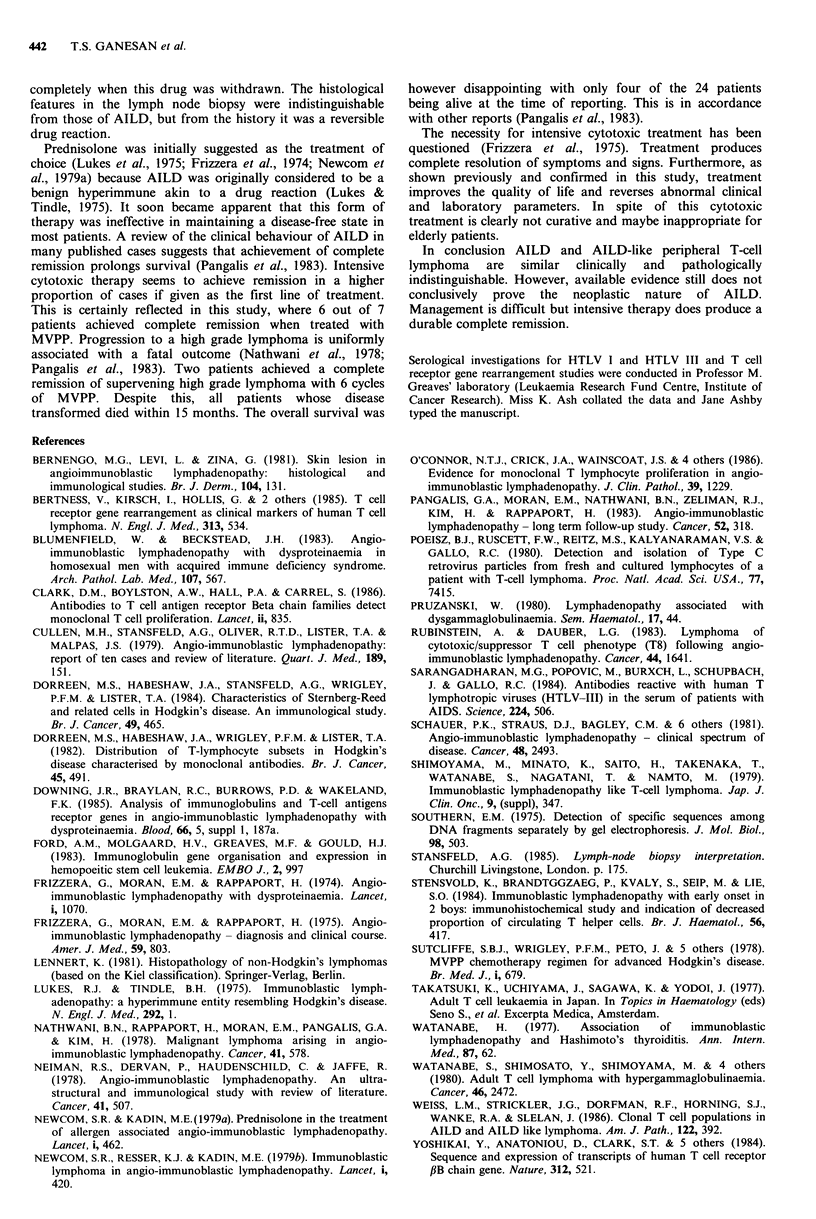# Angio-immunoblastic lymphadenopathy: a clinical, immunological and molecular study.

**DOI:** 10.1038/bjc.1987.86

**Published:** 1987-04

**Authors:** T. S. Ganesan, H. S. Dhaliwal, M. S. Dorreen, A. G. Stansfeld, J. A. Habeshaw, T. A. Lister

## Abstract

Twenty four patients with angio-immunoblastic lymphadenopathy (AILD) presenting between 1974 and 1985 have been reviewed. Clinical features at presentation included rash, fever, lymphadenopathy and hepatosplenomegaly in 75% of patients. Polyclonal hypergammaglobulinaemia was seen in 19/20 patients; 5 had normal immunoglobulin levels. Combination chemotherapy with MVPP was the optimal treatment with 6/7 patients achieving complete remission. Duration of remission ranged from 9 months to 4 years and was significantly longer in patients achieving complete as opposed to partial remission. In 6 patients phenotype studies were performed on single cell suspensions and immunoperoxidase studies on frozen sections of 7 lymph nodes. There was a reversal of the normal T suppressor/helper cell ratio with a predominance of T suppressor cells. Loss of normal B follicles was observed histologically in all except one lymph node. Germline configuration of the beta B-chain of the T cell receptor was observed in lymph nodes of 4 patients with AILD, and a rearranged T cell receptor was observed in 1 patient in whom a second lymph node biopsy had shown alteration of the histological picture to that of T-zone lymphoma. Frozen sera of 15 patients were screened for antibodies to HTLV I and III and were found to be negative.


Br. J. Cancer (1987), 55, 437–442
© The Macmillan Press Ltd., 1987

### Angio-immunoblastic lymphadenopathy: A clinical, immunological and molecular study

T.S. Ganesan[1], H.S. Dhaliwal[2], M.S. Dorreen[3], A.G. Stansfeld[1], J.A. Habeshaw[1] & T.A. Lister[1]

[1]*ICRF Department of Medical Oncology, St. Bartholomew's Hospital, London, EC1A,* [2]*Leukaemia Research Fund Centre, Institute of Cancer Research, Fulham Road, London, SW3 and* [3]*Department of Medicine, Royal Hallamshire Hospital, Sheffield, UK.*

**Summary** Twenty four patients with angio-immunoblastic lymphadenopathy (AILD) presenting between 1974 and 1985 have been reviewed. Clinical features at presentation included rash, fever, lymphadenopathy and hepatosplenomegaly in 75% of patients. Polyclonal hypergammaglobulinaemia was seen in 19/20 patients; 5 had normal immunoglobulin levels. Combination chemotherapy with MVPP was the optimal treatment with 6/7 patients achieving complete remission. Duration of remission ranged from 9 months to 4 years and was significantly longer in patients achieving complete as opposed to partial remission.

In 6 patients phenotype studies were performed on single cell suspensions and immunoperoxidase studies on frozen sections of 7 lymph nodes. There was a reversal of the normal T suppressor/helper cell ratio with a predominance of T suppressor cells. Loss of normal B follicles was observed histologically in all except one lymph node. Germline configuration of the βB-chain of the T cell receptor was observed in lymph nodes of 4 patients with AILD, and a rearranged T cell receptor was observed in 1 patient in whom a second lymph node biopsy had shown alteration of the histological picture to that of T-zone lymphoma.

Frozen sera of 15 patients were screened for antibodies to HTLV I and III and were found to be negative.

Angio-immunoblastic lymphadenopathy with dysproteinaemia (AILD) is a clinicopathological syndrome originally described in 1974 (Frizzera *et al.*, 1974; Lukes & Tindle, 1975). The pathogenesis of this condition is still not understood clearly, although knowledge of the clinical features, natural history and management of the disease have improved considerably. The disease was initially believed to be a 'hyperimmune phenomenon' which was in some instances, triggered by exposure to drugs to which the patient had become sensitised (Lukes & Tindle, 1975; Cullen *et al.*, 1979). In most cases however the nature of the supposed allergen was obscure. It was only recognised that a proportion of cases progressed to malignant lymphoma usually of high grade, immunoblastic type (Lukes & Tindle, 1975; Nathwani *et al.*, 1978). Although many clinicians at that time felt that the clinical behaviour of the lesion was from the onset more in keeping with a malignant neoplasm, pathologists in general were reluctant to accept that the process was neoplastic in the early stages. It was widely believed that AILD was pre-malignant, and not malignant. At the end of the decade, Shimoyama *et al.*, 1979 and Watanabe *et al.*, 1980, described a form of peripheral T-cell lymphoma which was clinically and pathologically indistinguishable from AILD, as it has been described in the American and European literature. Since that time, the theory that AILD is, at least in the majority of cases, a special type of T-cell lymphoma, has steadily gained ground, although many of the curious features of this disease remain unexplained.

This report presents the cumulative experience of patients studied prospectively since the first report from St. Bartholomew's Hospital (Cullen *et al.*, 1979), with analysis of lymphocyte subpopulations in 6 patients by immunohistological techniques using monoclonal antibodies. The clonality of T cells has also been evaluated by analysis of T cell receptor gene rearrangements in 4 patients in whom tissue was available.

### Materials and methods

### *Patients*

Twenty-six patients with AILD, of whom 10 formed the basis of the previous report (Cullen *et al.*, 1979) were referred to St. Bartholomew's Hospital between March 1974 and April 1985. All patients in whom the diagnosis of AILD was made between 1974–1985 were reviewed histologically. One patient was excluded as the diagnosis was changed to reactive lymphadenitis on review of the biopsied section. Follow up data were not available in another. The diagnosis was confirmed in all patients by lymph node biopsy. Serial lymph node biopsies were undertaken if there was progression of disease on treatment, or at relapse. Routine assessment included biochemical screening, computed tomographic (CT) scans of abdomen and chest, and bone marrow aspirate and biopsy. After the extent of disease had been defined, chemotherapy was commenced and disease status reassessed on completion of treatment.

### *Immunopathological studies*

Lymphocytes were isolated from peripheral blood by centrifugation on Ficoll-Triosil gradients and single cell suspensions were prepared from excised lymph nodes or spleen. For the immunohistological studies, sections (5 $\mu$m) were cut from frozen blocks of tissue stored in the gas phase of liquid nitrogen, and immunostained by the avidin-biotin conjugate method (ABC technique) and immunoperoxidase technique as described by Dorreen *et al.*, (1982, 1984).

Phenotypic analysis of single cell suspensions obtained in this way was performed by the indirect immunofluorescent assay using primary monoclonal antisera and fluoresceinated F(ab) goat anti-mouse IgG as secondary antibody. Surface membrane immunoglobulin expression was determined using heterologous class specific antisera in the direct immunofluorescence technique (Dorreen *et al.*, 1984).

### *Molecular studies*

DNA was prepared from frozen lymph nodes, digested with restriction enzymes (ECOR1, BAMH1, HINDIII and ECORV) and transferred to nitrocellulose by the technique of Southern (1975). The samples were hybridised using the T-cell receptor probe CB1 (Yoshikai *et al.*, 1984) and the immunoglobin gene $J_H$ probe as described previously (Ford *et al.*, 1983).

### *HTLV I and HTLV III serology*

Sera from 15 patients stored at −70°C were screened for

Correspondence: T.A. Lister.
Received 1 July 1986: and in revised form, 15 December 1986.

antibodies against retroviruses HTLV I and HTLV III by the Elisa technique (Sarangadharan *et al.*, 1984).

### Results

### *Clinical features*

The age of patients ranged between 42 and 77 years (median 53 years) except one patient who was 10 years of age.

All patients (14 men and 10 women) presented with lymphadenopathy and other features as shown in Table I. In one patient the disease appeared to have been precipitated by a drug (Maloprim) and the symptoms subsided on withdrawal of the drug and did not recur. Six other patients developed drug related rashes during the initial presentation. In addition to the typical features of AILD, ascites was noted in 4 patients and pedal oedema in eight. Dyspnoea associated with radiologically small pleural effusions was observed in 5 patients and lung function tests in two of them showed a restrictive type of defect. Four patients had transient polyarthritis without radiological changes, and bilateral carpal tunnel syndrome was observed in one patient. Enlarged lymph nodes caused dysphagia in one patient.

**Table I** Clinical features

| *Clinical features* | *(Total patients = 24)* *No. of patients* |
|---|---|
| Fever | 18 |
| Lymphadenopathy | |
| a) generalised | 15 |
| b) localised | 7 |
| Hepatomegaly | 18 |
| Splenomegaly | 16 |
| Arthritis | 5 |
| Ascites | 4 |
| Oedema | 8 |
| Dermatological features | |
| a) non-specific rash | 18 |
| b) purpura | 3 |
| c) nodules | 2 |

### *Haematological profile* (Table II)

Anaemia was noted in 17/24 patients, 9 of whom had a positive Coombs test, 5 with reticulocytosis. Leucocytosis ($>11\times10^9\,l^{-1}$) was present in 12/24 and eosinophilia was prominent in 9/24 while 15 patients had lymphopenia ($<1.5\times10^9\,l^{-1}$). Circulating atypical mononuclear cells were observed in 9 patients. These abnormal features returned to normal during remission in all patients.

Bone marrow examination was normal at presentation in 7/17 patients. The other ten patients had marrow involvement characterised by a polymorphous infiltrate of plasma cells, immunoblasts, lymphoplasmacytoid cells and eosinophils. Trephine biopsies revealed an increase in reticulin and vessels.

**Table II** Haematological features

| | |
|---|---|
| Anaemia ($<10\,g\,l^{-1}$) | 17/24 |
| Leucocytosis ($>11\times10^9\,l^{-1}$) | 12/24 |
| Eosinophilia ($>0.44\times10^9\,l^{-1}$) | 9/24 |
| Atypical cells in peripheral blood | 11/24 |
| Thrombocytopenia ($<150\times10^9\,l^{-1}$) | 7/24 |
| Coombs test positivity | 9/24 |
| Lymphocytopenia ($<1.5\times10^9\,l^{-1}$) | 15/24 |
| ESR (Westergren $>150\,mm\,h^{-1}$) | 9/20 |

### *Biochemical features* (Table III)

Hyponatremia ($<135\,mmol\,l^{-1}$) at presentation was noted in 12/23 patients. It reverted to normal during remission. Although hepatomegaly was observed in 18 patients, the liver function tests were normal in all except 3 who had hypoalbuminaemia. Enzyme studies showed hydroxybutyrate dehydrogenase (a nonspecific test measuring lactate dehydrogenase enzyme levels) to be elevated in 16/17. Five patients had biochemical evidence of hypothyroidism but were clinically euthyroid. Two of these had antithyroid autoantibodies. Protein electrophoresis showed hypergammaglobulinaemia in 14 patients, a normal pattern in 5 and hypogammaglobulinaemia in one.

**Table III** Biochemical features

| | |
|---|---|
| Hypoalbuminaemia ($<34\,g\,l^{-1}$) | 3/24 |
| Hyponatraemia ($<135\,mmol\,l^{-1}$) | 12/23 |
| Elevated HBD (normal 40–125 IU) | 16/17 |
| Abnormal thyroid function tests indicating hypothyroidism | 5/16 |
| Hypergammaglobulinaemia | 14/20 |
| Normal immunoglobulin electrophoresis | 5/20 |
| Hypogammaglobulinaemia | 1/20 |

### *Immunopathological features*

Fresh lymph nodes were available only in 6/24 patients. Studies on single cell suspensions in 7 lymph node biopsies from 6 patients at presentation showed predominance of T cells in all except one (Table IV). The majority of T cells were of the 'suppressor' phenotype (OKT8+ve), resulting in either a marked reduction, or reversal of the helper to suppressor T cell ratio ($T_h$:$T_s$ – normal range 3–4:1). Polyclonal B cells (SIg+ve) were present in all 4 nodes tested. The proportion of B cells was variable and correlated with HLA-DR positive cells.

The ratio of $T_h$:$T_s$ in the peripheral blood was reversed in 2 out of 3 patients in whom the disease had altered to a T-zone lymphoma at the time of testing, whilst the $T_h$:$T_s$ ratio was within normal limits in the third patient with AILD (untransformed).

### *Immunoperoxidase studies* (Table V)

Immunoperoxidase studies on frozen sections of the same nodes showed the overall preponderance of T cells and confirmed the reversal of $T_h$:$T_s$ ratio observed in single cell suspension studies of 2 patients. In 3 other patients only a reduction of $T_h$:$T_s$ ratio was noted (1.5 to 2.0). The T cells were distributed as a diffuse infiltrate over the entire lymph node. Natural killer cells (detected by reaction with the Leu 7 antibody) were increased (10–30%) in 2/5 nodes compared with normal or reactive nodes and did not show the usual localisation in follicles or follicle-like structures. In 1 patient (GR) increased proportions of Leu 7+ve cells were still evident in blood and lymph node tissue at the time of transformation into T-zone lymphoma. The absence of residual B cell follicles and a diffuse T cell infiltrate were prominent features in all but one case of AILD. Reaction with OKT9 (transferrin receptor antibody) was not increased in three lymph nodes tested and in one patient the germinal centre cells were positive. Only the large blast like T cells were OKT9 positive in one lymph node with features of T-zone lymphoma.

### *Serology for HTLV I and III*

Screening on frozen sera of 15 patients for antibodies against HTLV I and III was performed and was found to be negative in all cases.

**Table IV** Phenotypic studies of single cell suspensions. (All figures are expressed as percentages of total viable mononuclear cells)

| *Patient* | *E. Rosettes + ve cells* | *Pan T** | *Th (OKT4/Leu 3a)* | *Ts (OKT8-Leu 2a)* | *Th:Ts* | *OKT9* | *(smIg)* | *Ia* |
|---|---|---|---|---|---|---|---|---|
| A: *Lymph Node* | | | | | | | | |
| 1 JP | 18 | 27 | 22.5 | 11.5 | 2:1 | 6 | 27 (polyclonal) | 21 |
| 2 DD | 67 | 57 | — | 38 | NT or <1 | — | — | 40 |
| 3 GR | 61 | 94 | 29 | 64 | 1:2 | — | — | 4 |
| 4 WP | 57 | >95 | 36 | 73 | 1:2 | 6 | 1–2 (polyclonal) | 27 |
| 5 IM (1980) | 50 | 70 | 15 | 27 | 1:2 | — | 29 (polyclonal) | — |
| (1981) | 14 | 30 | 2 | 9 | 1:4.5 | — | 46 (polyclonal) | — |
| 6 EP | — | 60 | 34 | 27 | 1.3:1 | — | 10 (polyclonal) | — |
| B. *Blood* | | | | | | | | |
| 1 DD | — | 45 | 70 | 51 | 4.7:1 | — | — | 30 |
| 2 GR | — | 79 | 12 | 64 | 1:5 | 0 | B1-2 | 1 |
| 3 WP | 50 | 89 | 26 | 62 | 1:2.4 | — | — | 10 |
| *Reactivity with OKT1 or OKT3. | | | | | | | | |
| Controls: | | | | | | | | |
| Mean ± | 39 | 55 | 47 | 1 | 4:1 | <5 | 16 | 23 |
| s.e. | 6 | 6 | 3 | 1 | | | 3 | 2 |

**Table V** Immunoperoxidase studies of the distribution of lymphocyte subpopulations in involved lymph nodes

| *Patient* | *T cell reaction* | *T4/Leu 3a (%)* | *T8/Leu (%)* | *Th:Ts* | *Leu-7* | *OKT9* | *OKM/1 Mo-2* |
|---|---|---|---|---|---|---|---|
| IM | ++/+++ | 70 | 30 | 2:1 | <10%: focal distribution | neg | <1% |
| DD | +++ | 15 | 86 | 1:6 | <1% | neg | 15–25% |
| GR | +++/++++ | 30 | 70 | 1:2 | 30%: even distribution | some +ve larger cells | — — |
| RP | +++ | 60 | 40 | 1.5:1 | 10–30% marked interfollicular reaction. Also germinal ctrs & mantle zones | neg | — |
| PB | +++ | 60 | 40 | 1.5:1 | <1% | diffuse stain of mo's | 20–30% |
| GR (NHL) | ++++ | 60 | 40 | 1.5:1 | 10–30%: as for RP | germ ctr. cells, mo's & lymphoma cells | 10–15% |

++ = moderate +++ = marked ++++ = intense
Note: All percentages quoted are approximate estimates.

### *Molecular analysis of immunoglobulin and T cell receptor genes*

Analysis of $\beta$B chain of the T cell receptor (CB1) and immunoglobulin genes for rearrangement showed no evidence of a clonal population of T or B cells in lymph nodes of 4 patients with AILD in whom frozen lymph node tissue was available. However in one patient whose disease had evolved to a T-zone lymphoma, there was clonal rearrangement of the $\beta$B chain of the T cell receptor gene.

### *Clinical course* (Table VI)

Five patients received no therapy initially and 2 of these had prolonged freedom from disease after a spontaneous remission, for 4 and 6 years respectively. Prednisolone was used alone in 3 patients with 1 patient achieving a complete remission lasting 2 years. Cyclophosphamide and prednisolone were given to 8 patients as initial therapy. Only 1 patient had a complete remission, with 4 partial responses; the rest failed to respond. Six out of seven patients receiving combination chemotherapy comprising mustine, vinblastine, prednisolone and procarbazine (MVPP) (Sutcliffe *et al.*, 1978) achieved complete remission. The duration of remission ranged from 9 months to 37 months (median 4 months). Five patients developed overt non Hodgkin's lymphoma (T-zone lymphoma in 2 patients and immunoblastic lymphoma in 3 patients) (Table VII). This was evident on lymph node biopsy during relapse or progression of disease. The trans-

**Table VI** Overall response to treatment

| *Initial treatment* | *No. of pts.* | *No. of pts.* CR | PR/fail | *Duration of remission (months)* Range | Median |
|---|---|---|---|---|---|
| MVPP | 7 | 6 | 1 | 9–37 | 24 |
| Cyclophosphamide and prednisolone | 8 | 1 | 7 | 9 | 9 |
| Prednisolone | 3 | 1 | 2 | 26 | 26 |

**Table VII** Details of patients transforming to a high grade lymphoma

| *Pt. No.* | *Response to therapy for AILD* | *Duration of remission (months)* | *Histology of transformed lymphoma* | *Response to therapy after transformation* | *Survival after diagnosis of transformation* |
|---|---|---|---|---|---|
| 1 | CR | 24 | T zone lymphoma | not assessable | 8 |
| 2 | Fail | 4 | T zone lymphoma | not assessable | 11 |
| 3 | CR | 71 | IL | CR | 15 |
| 4 | Fail | 6 | IL | NR | 6 |
| 5 | PR | 21 | IL | CR | 7 |

formation was not associated with any change in clinical behaviour. Although complete remission of the lymphoma was achieved with combination therapy in 2 patients, it was followed by early relapse and all these patients died within a year.

The overall survival for all the patients is as shown in Figure 1. Survival was longer in patients achieving complete remission ($P<0.03$) (Figure 2).

Autopsy details are available in 7 patients (Table VIII). The cause of death appeared to be related to immunosuppression in 3 patients. Two patients had cytomegalovirus in the lungs, and one had pneumocystis pneumonia. Immunoblastic lymphoma was diagnosed at post mortem in one patient.

**Figure 1** Overall survival of AILD patients.

**Figure 2** Overall survival of AILD patients according to response to therapy.

### Discussion

The clinical features of AILD at presentation in this series are similar to those reported in the literature (Frizzera *et al.*, 1974; Lukes & Tindle, 1975; Schauer *et al.*, 1981; Pruzanski, 1980), namely rapid onset of constitutional symptoms with rash, generalised lymphadenopathy, and hepatosplenomegaly. Dermatological manifestations in three-quarters of the patients consisted of a non-specific papular rash. Three patients presented with purpura, and 2 had nodular skin lesions, as described by Bernengo *et al.*, (1981), with histological features of AILD on skin biopsy. Haemolytic anaemia (HA) was a presenting feature in a

**Table VIII** Summary of post mortem findings

| *Pt. No.* | *AILD* | *LYMPHOMA* | *PNEUMONIA* | *UNRELATED* |
|---|---|---|---|---|
| 1 | + | — | — | Haematemesis splenic rupture |
| 2 | — | + in lymph nodes | — | Peritonitis |
| 3 | — | — | + | Myocardial infarction |
| 4 | + | — | + | — |
| 5 | Fibrosis with lymphocyte depletion in lymph nodes | — | Cytomegalovirus pneumocystis | Myocardial infarction Pulmonary infarct |
| 6 | — | Lymphoma | — | Diverticulitis silent perforation of pelvic colon |
| 7 | Hyalin tissue in lymph nodes | — | Cytomegalovirus | Ulcer first part of duodenum |

quarter of the patients with a positive Coombs reaction, exhibiting IgG and C3d on the red cells. A lower incidence of HA has been reported by others (Neiman *et al.*, 1978; Pangalis *et al.*, 1978).

Although hypergammaglobulinaemia was an integral part of the AILD syndrome as first described, (Frizzera *et al.*, 1974; Lukes & Tindle, 1975), five out of 24 patients in this series had normal immunoglobulin levels at presentation, which later became elevated, and one patient presented with hypogammaglobulinaemia. Hypothyroidism with autoantibodies is not a common feature in previous reports (Watanabe *et al.*, 1977). In this study, 5 out of 24 patients had laboratory evidence of thyroid dysfunction whilst clinically euthyroid.

The histopathological features of AILD in a lymph node biopsy are distinctive despite the uncertainty which surrounds their interpretation. The normal architecture of the node is effaced, with disappearance of follicles and gross reduction of typical small lymphocytes which are replaced by a polymorphic infiltrate of immunoblasts, plasma cells eosinophils, histiocytes and atypical lymphocytes with irregularly shaped nuclei which mark as T cells on immunostaining. Mitoses are sometimes numerous. A prominent feature is the arborising vascular proliferation which accompanies the infiltration and in some instances the deposition of amorphous eosinophilic material. (Lukes & Tindle, 1975; Frizzera *et al.*, 1974).

Clinically and pathologically AILD has many features suggestive of an abnormal immune reaction. There is evidence of an abnormal sensitivity to drugs in some patients, the frequent occurrence of skin rashes, and less frequently arthropathy; also a tendency to spontaneous remissions, especially in the early stages of the disease. Pathologically the observation of polyclonal hypergammaglobulinaemia in most cases, the frequent occurrence of autoimmune phenomenona (Coombs-positive haemolytic anaemia, thrombocytopenia) and alteration or reversal of the $T_h/T_s$ ratio, all indicate an immune disturbance. Delayed hypersensitivity tests may show cutaneous anergy to common antigens implying dysfunction of T-helper/inducer function. Frizzera *et al.* (1974) likened the process to a graft versus host reaction, whilst Lukes and Tindle (1975) raised the possibility that certain drugs might give rise to a prolonged and disordered hypersensitivity reaction. Because of the often marked plasma cell infiltration, immunoblastic proliferation and the almost invariable polyclonal hypergammaglobulinaemia, a primary disorder of the B cell lineage was earlier suspected, (Lukes & Tindle, 1975). The frequent transformation of AILD to immunoblastic lymphoma (Nathwani *et al.*, 1978; Newcom *et al.*, 1979b; Cullen *et al.*, 1979) has also been cited as supportive evidence of a B cell disorder.

The more recent observations of T cell subsets alteration has directed attention away from B cells to that of T cells. In this study, a reversal of $T_h/T_s$ ratio and an absolute increase in T cells was observed in cell suspensions of lymph nodes from 6 patients and similar findings have been reported in the peripheral blood lymphocytes in two young males with AILD (Stensvold *et al.*, 1984). In this regard, the similarity to the findings to HIV infection are noteworthy, and prompted investigation of whether HTLV I or HTLV III play an aetiological role in AILD (Poeisz *et al.*, 1980; Takatsuki *et al.*, 1977). Recently there was a report describing an association of AILD and acquired immunodeficiency syndrome (AIDS) in two patients (Blumenfield *et al.*, 1983). Frozen sera from 15 patients in the present series were tested and found to be negative for antibodies to these viruses.

That AILD may transform into a high grade lymphoma is well known, but the phenotype has been established only in a few cases. Lennert (1981) recorded the transformation of a case of AILD into a T-zone lymphoma and the same sequence was observed in 2 cases of the present series. One termination of AILD as a T-cell lymphoma of $T_8$(suppressor) phenotype has been recorded (Rubenstein & Dauber, 1983). Only in the last few years has the proposition been advanced that many cases of AILD may in fact be T-cell lymphomas from the outset. This was first suggested by a group of Japanese workers (Shimoyama *et al.*, 1979; Watanabe *et al.*, 1980) who desribed a series of immunoblastic lymphadenopathy-like T-cell lymphomas with clinical and pathological features similar to AILD, including the occurrence of polyclonal hypergammaglobulinaemia. In addition to the behaviour of the disease in their patients, these authors adduced the finding of karyotypic abnormalities as evidence for the neoplastic nature of the disease.

The idea that AILD might, in many instances at least, be a special type of peripheral T-cell lymphoma, has led in the past few years to a reappraisal of the clinical and pathological features of this condition. With increased recognition of the special peculiarities of peripheral T-cell lymphomas the idea no longer seems improbable, as it did 10 years ago. Indeed several of the histological features of AILD are shared by other recognised varieties of peripheral T-cell lymphoma. In view of the acknowledged tendency in T-cell lymphomas for the histological picture to change between one biopsy and the next (Stansfeld, 1985) it is not surprising that the picture of AILD should sometimes change into that of a T-zone lymphoma.

Further support for the neoplastic nature of AILD has come from 2 sources. First, karyotypic abnormalities have been shown in lymph nodes from patients with AILD. Secondly, clonal rearrangement of the $\beta$ chain on the T cell receptor gene has been shown in such lymph nodes. Whilst clonal rearrangement does not constitute final proof of the neoplastic nature of the disease it is certainly suggestive of neoplasia. The results are not, however, entirely consistent. In a series of 24 patients with AILD, O'Connor and Mason (1986), showed clonal rearrangements of the $\beta$ chain of the T cell receptors only in 2/3. Similar results were reported by Bertness *et al.* (1985), Downing *et al.*, (1985) and Weiss *et al.* (1986). Further, it was shown in the above reports that 2 patients showed T cell clonality only when the disease evolved into a definite T-cell lymphoma. In the present study, none of the four patients showed evidence of T cell clonality in the first biopsy. In conformity with the reports cited above, however, rearrangement was found in one patient after his disease had evolved into a T-zone lymphoma.

Whilst it is still possible that AILD showing the germline pattern of the T cell receptor gene is neoplastic (the clonal population may be too small for detection), it is also possible that AILD in its initial stages is preneoplastic, as suggested earlier. Such a theory prompts the question as to whether it is possible to distinguish histologically between premalignant and malignant stages of the disease, which seems almost invariably to declare itself as become malignant in course of time. In a recent study, cited above, (Weiss *et al.*, 1986), the authors attempted to distinguish morphologically between AILD and AILD-like lymphoma in a series of 10 patients. T cell clonality was found in three out of five specimens from patients whose biopsies were interpreted as showing AILD and in five out of six specimens where the diagnosis was AILD-like lymphoma. It thus seems fairly clear that 'premalignant' and malignant phases of AILD cannot be reliably distinguished morphologically. A recent study (Clark *et al.*, 1986) holds out hope that a simpler method of assessing T cell clonality may be available in the near future. Few would doubt that the established disease is, in the great majority of cases, a T-cell lymphoma.

Only one patient in the present series (Case 11 of the previous report – Cullen *et al.*, 1979) seems to be different in that his disease was clearly due to sensitisation to a drug – Maloprim (Dapsone and Pyrimethamine), taken for malarial prophylaxis. Lymphadenopathy and rash recurred every time this drug was taken and resolved subsequently. He recovered completely when this drug was withdrawn. The histological features in the lymph node biopsy were indistinguishable from those of AILD, but from the history it was a reversible drug reaction.

Prednisolone was initially suggested as the treatment of choice (Lukes *et al.*, 1975; Frizzera *et al.*, 1974; Newcom *et al.*, 1979a) because AILD was originally considered to be a benign hyperimmune akin to a drug reaction (Lukes & Tindle, 1975). It soon became apparent that this form of therapy was ineffective in maintaining a disease-free state in most patients. A review of the clinical behaviour of AILD in many published cases suggests that achievement of complete remission prolongs survival (Pangalis *et al.*, 1983). Intensive cytotoxic therapy seems to achieve remission in a higher proportion of cases if given as the first line of treatment. This is certainly reflected in this study, where 6 out of 7 patients achieved complete remission when treated with MVPP. Progression to a high grade lymphoma is uniformly associated with a fatal outcome (Nathwani *et al.*, 1978; Pangalis *et al.*, 1983). Two patients achieved a complete remission of supervening high grade lymphoma with 6 cycles of MVPP. Despite this, all patients whose disease transformed died within 15 months. The overall survival was however disappointing with only four of the 24 patients being alive at the time of reporting. This is in accordance with other reports (Pangalis *et al.*, 1983).

The necessity for intensive cytotoxic treatment has been questioned (Frizzera *et al.*, 1975). Treatment produces complete resolution of symptoms and signs. Furthermore, as shown previously and confirmed in this study, treatment improves the quality of life and reverses abnormal clinical and laboratory parameters. In spite of this cytotoxic treatment is clearly not curative and maybe inappropriate for elderly patients.

In conclusion AILD and AILD-like peripheral T-cell lymphoma are similar clinically and pathologically indistinguishable. However, available evidence still does not conclusively prove the neoplastic nature of AILD. Management is difficult but intensive therapy does produce a durable complete remission.

Serological investigations for HTLV I and HTLV III and T cell receptor gene rearrangement studies were conducted in Professor M. Greaves' laboratory (Leukaemia Research Fund Centre, Institute of Cancer Research). Miss K. Ash collated the data and Jane Ashby typed the manuscript.

**References**

BERNENGO, M.G., LEVI, L. & ZINA, G. (1981). Skin lesion in angioimmunoblastic lymphadenopathy: histological and immunological studies. *Br. J. Derm.*, **104**, 131.

BERTNESS, V., KIRSCH, I., HOLLIS, G. & 2 others (1985). T cell receptor gene rearrangement as clinical markers of human T cell lymphoma. *N. Engl. J. Med.*, **313**, 534.

BLUMENFIELD, W. & BECKSTEAD, J.H. (1983). Angio-immunoblastic lymphadenopathy with dysproteinaemia in homosexual men with acquired immune deficiency syndrome. *Arch. Pathol. Lab. Med.*, **107**, 567.

CLARK, D.M., BOYLSTON, A.W., HALL, P.A. & CARREL, S. (1986). Antibodies to T cell antigen receptor Beta chain families detect monoclonal T cell proliferation. *Lancet*, **ii**, 835.

CULLEN, M.H., STANSFELD, A.G., OLIVER, R.T.D., LISTER, T.A. & MALPAS, J.S. (1979). Angio-immunoblastic lymphadenopathy: report of ten cases and review of literature. *Quart. J. Med.*, **189**, 151.

DORREEN, M.S., HABESHAW, J.A., STANSFELD, A.G., WRIGLEY, P.F.M. & LISTER, T.A. (1984). Characteristics of Sternberg-Reed and related cells in Hodgkin's disease. An immunological study. *Br. J. Cancer*, **49**, 465.

DORREEN, M.S., HABESHAW, J.A., WRIGLEY, P.F.M. & LISTER, T.A. (1982). Distribution of T-lymphocyte subsets in Hodgkin's disease characterised by monoclonal antibodies. *Br. J. Cancer*, **45**, 491.

DOWNING, J.R., BRAYLAN, R.C., BURROWS, P.D. & WAKELAND, F.K. (1985). Analysis of immunoglobulins and T-cell antigens receptor genes in angio-immunoblastic lymphadenopathy with dysproteinaemia. *Blood*, **66**, 5, suppl 1, 187a.

FORD, A.M., MOLGAARD, H.V., GREAVES, M.F. & GOULD, H.J. (1983). Immunoglobulin gene organisation and expression in hemopoeitic stem cell leukemia. *EMBO J.*, **2**, 997

FRIZZERA, G., MORAN, E.M. & RAPPAPORT, H. (1974). Angio-immunoblastic lymphadenopathy with dysproteinaemia. *Lancet*, **i**, 1070.

FRIZZERA, G., MORAN, E.M. & RAPPAPORT, H. (1975). Angio-immunoblastic lymphadenopathy – diagnosis and clinical course. *Amer. J. Med.*, **59**, 803.

LENNERT, K. (1981). Histopathology of non-Hodgkin's lymphomas (based on the Kiel classification). Springer-Verlag, Berlin.

LUKES, R.J. & TINDLE, B.H. (1975). Immunoblastic lymph-adenopathy: a hyperimmune entity resembling Hodgkin's disease. *N. Engl. J. Med.*, **292**, 1.

NATHWANI, B.N., RAPPAPORT, H., MORAN, E.M., PANGALIS, G.A. & KIM, H. (1978). Malignant lymphoma arising in angio-immunoblastic lymphadenopathy. *Cancer*, **41**, 578.

NEIMAN, R.S., DERVAN, P., HAUDENSCHILD, C. & JAFFE, R. (1978). Angio-immunoblastic lymphadenopathy. An ultra-structural and immunological study with review of literature. *Cancer*, **41**, 507.

NEWCOM, S.R. & KADIN, M.E. (1979*a*). Prednisolone in the treatment of allergen associated angio-immunoblastic lymphadenopathy. *Lancet*, **i**, 462.

NEWCOM, S.R., RESSER, K.J. & KADIN, M.E. (1979*b*). Immunoblastic lymphoma in angio-immunoblastic lymphadenopathy. *Lancet*, **i**, 420.

O'CONNOR, N.T.J., CRICK, J.A., WAINSCOAT, J.S. & 4 others (1986). Evidence for monoclonal T lymphocyte proliferation in angio-immunoblastic lymphadenopathy. *J. Clin. Pathol.*, **39**, 1229.

PANGALIS, G.A., MORAN, E.M., NATHWANI, B.N., ZELIMAN, R.J., KIM, H. & RAPPAPORT, H. (1983). Angio-immunoblastic lymphadenopathy – long term follow-up study. *Cancer*, **52**, 318.

POEISZ, B.J., RUSCETT, F.W., REITZ, M.S., KALYANARAMAN, V.S. & GALLO, R.C. (1980). Detection and isolation of Type C retrovirus particles from fresh and cultured lymphocytes of a patient with T-cell lymphoma. *Proc. Natl. Acad. Sci. USA.*, **77**, 7415.

PRUZANSKI, W. (1980). Lymphadenopathy associated with dysgammaglobulinaemia. *Sem. Haematol.*, **17**, 44.

RUBINSTEIN, A. & DAUBER, L.G. (1983). Lymphoma of cytotoxic/suppressor T cell phenotype (T8) following angio-immunoblastic lymphadenopathy. *Cancer*, **44**, 1641.

SARANGADHARAN, M.G., POPOVIC, M., BURXCH, L., SCHUPBACH, J. & GALLO, R.C. (1984). Antibodies reactive with human T lymphotropic viruses (HTLV–III) in the serum of patients with AIDS. *Science*, **224**, 506.

SCHAUER, P.K., STRAUS, D.J., BAGLEY, C.M. & 6 others (1981). Angio-immunoblastic lymphadenopathy – clinical spectrum of disease. *Cancer*, **48**, 2493.

SHIMOYAMA, M., MINATO, K., SAITO, H., TAKENAKA, T., WATANABE, S., NAGATANI, T. & NAMTO, M. (1979). Immunoblastic lymphadenopathy like T-cell lymphoma. *Jap. J. Clin. Onc.*, **9**, (suppl), 347.

SOUTHERN, E.M. (1975). Detection of specific sequences among DNA fragments separately by gel electrophoresis. *J. Mol. Biol.*, **98**, 503.

STANSFELD, A.G. (1985). *Lymph-node biopsy interpretation.* Churchill Livingstone, London. p. 175.

STENSVOLD, K., BRANDTGGZAEG, P., KVALY, S., SEIP, M. & LIE, S.O. (1984). Immunoblastic lymphadenopathy with early onset in 2 boys: immunohistochemical study and indication of decreased proportion of circulating T helper cells. *Br. J. Haematol.*, **56**, 417.

SUTCLIFFE, S.B.J., WRIGLEY, P.F.M., PETO, J. & 5 others (1978). MVPP chemotherapy regimen for advanced Hodgkin's disease. *Br. Med. J.*, **i**, 679.

TAKATSUKI, K., UCHIYAMA, J., SAGAWA, K. & YODOI, J. (1977). Adult T cell leukaemia in Japan. In *Topics in Haematology* (eds) Seno S., *et al.* Excerpta Medica, Amsterdam.

WATANABE, H. (1977). Association of immunoblastic lymphadenopathy and Hashimoto's thyroiditis. *Ann. Intern. Med.*, **87**, 62.

WATANABE, S., SHIMOSATO, Y., SHIMOYAMA, M. & 4 others (1980). Adult T cell lymphoma with hypergammaglobulinaemia. *Cancer*, **46**, 2472.

WEISS, L.M., STRICKLER, J.G., DORFMAN, R.F., HORNING, S.J., WANKE, R.A. & SLELAN, J. (1986). Clonal T cell populations in AILD and AILD like lymphoma. *Am. J. Path.*, **122**, 392.

YOSHIKAI, Y., ANATONIOU, D., CLARK, S.T. & 5 others (1984). Sequence and expression of transcripts of human T cell receptor $\beta$B chain gene. *Nature*, **312**, 521.